# Visual Field Defects in Patients With Optic Nerve Head Drusen

**DOI:** 10.7759/cureus.51317

**Published:** 2023-12-30

**Authors:** Ana Rita Viana, Sara Pereira, Alberto Lemos, Rita Basto, Renato Correia Barbosa, Alexandre Reis da Silva

**Affiliations:** 1 Ophthalmology Department, Hospital Pedro Hispano, Unidade Local de Saúde de Matosinhos, Matosinhos, PRT

**Keywords:** automated perimetry, optical coherence tomography, functional impairment, anatomical impairment, visual field, optic nerve head drusen

## Abstract

Background

Optic nerve head drusen (ONHD) are acellular deposits in the optic nerve head, whose pathophysiology remains not fully understood. Most patients with ONHD have visual field (VF) defects. This study aims to describe the VF defects observed in patients with ONHD and to compare the anatomical and functional impairment between visible and buried ONHD.

Methods

Patients with ONHD were retrospectively studied. The retinal nerve fiber layer (RNFL) average thickness and the ganglion cell complex (GCC) average thickness were collected from optical coherence tomography data. Visual field index (VFI), mean deviation (MD), and pattern standard deviation (PSD) were collected from 30-2 standard automated perimetry. An abnormal VF test was defined as having a Glaucoma Hemifield Test outside normal limits and/or a PSD with a p-value<5%. Eyes with superficial or buried ONHD based on visibility by slit-lamp ophthalmoscopy were compared.

Results

Sixty-six eyes of 36 patients were included in the study. The mean age was 39.6 ± 2.5 years. Forty-nine eyes (81.7%) presented a VF defect: concentric VF constriction in 19 (38.8%), arcuate scotoma in 16 (32.7%), enlarged blind spot in 9 (18.4%), unspecific VF defect in 8 (16.3%), and nasal step in 3 (6.1%). Thirty-four eyes (51.5%) had superficial ONHD and 32 eyes (48.5%) had buried ONHD. Patients with superficial ONHD were significantly older (p<0.001) and presented a significantly lower VFI (p=0.010), lower MD (p=0.002), higher PSD (p<0.001), thinner GCC (p<0.001), and thinner RNFL (p<0.001) than patients with buried ONHD. VF defects were present in 90.6% of eyes with superficial ONHD and 71.4% of eyes with buried ONHD (p=0.113). The type of VF defects differed between groups (p=0.020).

Conclusions

Functional and structural impairment is more evident in eyes with superficial ONHD, maybe because the presence of calcification leads to greater axonal damage. Buried ONHD is more prevalent in younger patients, progressing to a superficial location and becoming calcified with age.

## Introduction

Optic nerve head drusen (ONHD) are acellular deposits located in front of the lamina cribrosa in the optic nerve head, whose pathophysiology remains not fully understood [1-3]. Some authors have hypothesized that the formation of ONHD is secondary to abnormal axoplasmic transport in the optic nerve head or abnormal axonal metabolism, with continuous calcification resulting from chronic axonal disruption [3,4]. There is an increased prevalence of ONHD in primary family members, which supports that ONHD can be inherited in an autosomal dominant pattern with incomplete penetrance [3,5]. Clinical associations include glaucoma, retinitis pigmentosa, Usher syndrome type 1, pseudoxanthoma elasticum and angioid streaks, β-thalassemia, and Alagille syndrome [5].

ONHD is more common in Caucasians and presents a slight predominance in the female gender [2]. Most cases are bilateral [3]. Some ONHDs are superficial and directly visible on fundoscopy, while others are deeper in the optic nerve head and difficult to detect, mimicking papilledema [1]. Buried ONHDs are more frequent in children [4]. Different methods can be used for the diagnosis of ONHD, including fundoscopy, fluorescein angiography, autofluorescence, monochromatic filters, scanning laser ophthalmoscope, computed tomography, optical coherence tomography (OCT), and ultrasonography [6]. B-scan ultrasonography is traditionally considered the gold standard for detecting calcified ONHD. More recently, the spectral-domain OCT and swept-source OCT have become the gold standard for the visualization of superficial and buried ONHD [7].

Although often benign and asymptomatic, most patients with ONHD have visual field (VF) defects [2,3,8]. Retinal nerve fiber layer (RNFL) thinning has also been described in patients with ONHD [9,10]. Peripapillary RNFL thickness seems to correlate with the anatomic location of ONHD and to influence the extent of VF loss [11].

This study aims to describe the VF defects observed in patients with ONHD and to compare the anatomical and functional impairment between visible and buried ONHD.

This article was previously presented as an oral communication at the 65th Portuguese Congress of Ophthalmology on December 2, 2022.

## Materials and methods

The present study was conducted by the tenets of the Declaration of Helsinki. This was a retrospective study based on the clinical records of patients with ONHD diagnosed on fundus examination, B-scan ultrasonography, and OCT, who were followed at our center between 2018 and 2022.

Collected data included age, gender, eye laterality, follow-up time, best corrected visual acuity (BCVA), spherical equivalent, and intraocular pressure (IOP). The type of ONHD was classified as superficial or buried according to visibility by slit-lamp ophthalmoscopy. Yellowish nodular formations visible by slit-lamp biomicroscopy in the optic nerve head were classified as superficial ONHD, while ONHD not visible by fundoscopy, but only by B-scan ultrasonography and OCT, were classified as buried ONHD. Cases with mixed classification were excluded. The RNFL average thickness and the ganglion cell complex (GCC) average thickness were collected from OCT data (Avanti RTVue XR®, Optovue Inc., Fremont, California, USA) to assess the structural damage. Visual field index (VFI), mean deviation (MD), and pattern standard deviation (PSD) were collected from the Swedish interactive thresholding algorithm (SITA) 30-2 standard automated perimetry (Humphrey Fielder Analyzer®, Carl Zeiss AG, Jena, Germany) to evaluate the functional damage. An abnormal VF test was defined as having a Glaucoma Hemifield Test (GHT) outside normal limits and/or a PSD with a p-value<5%, with acceptable reliability indices. Eyes with superficial or buried ONHD were compared regarding these parameters. Patients with ONHD maintained regular follow-up including repeated OCT and automated perimetry evaluation every 6 months until stabilization was observed, then at least annually. The most recent OCT and automated perimetry reports were used for analysis.

All statistical analysis was performed using Statistical Package for the Social Sciences (SPSS) software version 26.0 (IBM Corp., Armonk, NY, USA), and p-values <0.05 were considered statistically significant. Continuous variables were described through mean (M) ± standard deviation (SD), and categorical variables through absolute (n) and relative (%) frequencies. Parametric tests were applied after the normality of the sample was confirmed by the Kolmogorov-Smirnov test. An independent samples T-test was used for the comparison of two continuous variables. A chi-square test for independence with Yated Continuity Correction was used for the comparison of two categorical variables.

## Results

Sixty-six eyes of 36 patients with ONHD were included in our study, from which 4 patients (11.1%) were of pediatric age. The mean age was 39.6 ± 2.5 years old. Most patients presented bilateral disease (N=30, 83.3%). Superficial (N=34, 51.5%) and buried (N=32, 48.5%) ONHD were evenly distributed. Visual acuity was uncompromised in most patients, with a mean best-corrected visual acuity (BCVA) of 0.1 ± 0.1 logMAR. The mean IOP was within the normal range (16.7 ± 3.8 mmHg). None of the eyes presented glaucoma. Four eyes (6.1%) were under hypotensive eyedrops for neuroprotection. One patient had Feingold syndrome. No other ocular or systemic conditions typically associated were present. Other characterization parameters of the global sample are summarized in Table 1.

**Table 1:** Characteristics of the global sample of patients with ONHD. N (%) are depicted for categorical variables and M ± SD (range) for continuous variables. BCVA, best corrected visual acuity; D, diopters; GCC, ganglion cell complex; IOP, intraocular pressure; logMAR, logarithm of the minimum angle of resolution; M, mean; MD, mean deviation; N, absolute frequencies; ONHD, optic nerve head drusen; PSD, pattern standard deviation; RNFL, retinal nerve fiber layer; SD, standard deviation; VFI, visual field index; %, relative frequencies.

Variables	
Number of eyes	66
Number of patients	36
Age (years)	39.6 ± 2.5 (10-86)
Follow-up time (months)	47.8 ± 3.8 (12-160)
Gender: Male/Female	12/24 (33.3/66.7)
Unilateral/bilateral disease	6/30 (16.7/83.3)
Type of drusen: superficial/buried	34/32 (51.5/48.5)
Visual field defects: absent/present	11/49 (18.3/81.7)
BCVA (logMAR)	0.1 ± 0.1 (0.0-0.5)
Spherical equivalent (D)	-0.63 ± 2.43 ([-6.00]-[+3.88])
IOP (mmHg)	16.7 ± 3.8 (10-25)
VFI (%)	85.2 ± 18.2% (6-100)
MD (dB)	-8.5 ± 6.9 ([-30.7]-[-0.1])
PSD (dB)	6.1 ± 3.6 (1.5-12.9)
GCC thickness (µm)	86.4 ± 14.9 (60-117)
RNFL thickness (µm)	89.3 ± 22.7 (49-129)

Most patients presented VF defects (N=49, 81.7%). VF constriction was the most frequent type in patients with ONHD, which was present in 19 eyes (38.8%), followed by arcuate scotoma, which was present in 16 eyes (32.7%). Other VF defects identified are summarized in Table 2. Only 3 eyes (4.5%) presented a severely depressed VF.

**Table 2:** Types of visual field defects in patients with ONHD. N, absolute frequencies; ONHD, optic nerve head drusen; %, relative frequencies.

Visual field defect, N (%)	
Visual field constriction	19 (38.8)
Arcuate scotoma	16 (32.7)
Blind spot enlargement	9 (18.4)
Unspecific visual field defects	8 (16.3)
Nasal step	3 (6.1)

Eyes with superficial (group A) and buried (group B) ONHD were compared (Table 3). Group A presented a significantly older age (p<0.001), a lower absolute value of spherical equivalent (p=0.002), lower perimetric VFI and MD indices (p=0.010 and p=0.002, respectively), a higher perimetric PSD (p<0.001), an inferior GCC average thickness (p<0.001), and an inferior RNFL average thickness (p<0.001) when compared with group B. No significant differences were found in BCVA (p=0.079) and IOP (p=0.088) between the two types of ONHD.

**Table 3:** Comparison of eyes with superficial and buried ONHD based on the visibility by slit-lamp ophthalmoscopy. N (%) are depicted for categorical variables and M ± SD (range) for continuous variables. BCVA, best corrected visual acuity; GCC, ganglion cell complex; IOP, intraocular pressure; logMAR, logarithm of the minimum angle of resolution; M, mean; MD, mean deviation; N, absolute frequencies; ONHD, optic nerve head drusen; PSD, pattern standard deviation; RNFL, retinal nerve fiber layer; SD, standard deviation; VFI, visual field index; %, relative frequencies; *, statistical significance.

Variables	Group A, superficial drusen (N=34)	Group B, buried drusen (N=32)	p-value
Age (years)	47.6 ± 16.0 (20-71)	31.0 ± 20.3 (10-86)	p<0.001*
Present visual field defects	29 (90.6)	20 (71.4)	p=0.113
BCVA (logMAR)	0.1 ± 0.1 (0.0-0.5)	0.0 ± 0.1 (0.0-0.2)	p=0.079
Spherical equivalent (D)	+0.28 ± 1.71 ([-2.75]-[+3.88])	-1.66 ± 2.73 ([-6.00]-[+3.75])	p=0.002*
IOP (mmHg)	17.3 ± 4.4 (10-25)	15.5 ± 1.9 (12-19)	p=0.088
VFI (%)	79.7 ± 22.3 (6-100)	91.7 ± 8.7 (72-100)	p=0.010*
MD (dB)	-10.8 ± 7.8 ([-30.7]-[-3.3])	-5.7 ± 4.2 ([-12.8]-[-0.1])	p=0.002*
PSD (dB)	7.6 ± 3.4 (1.7-12.9)	4.3 ± 2.8 (1.5-11.0)	p<0.001*
GCC thickness (µm)	78.4 ± 15.0 (60-117)	95.6 ± 7.9 (77-105)	p<0.001*
RNFL thickness (µm)	79.0 ± 22.3 (49-128)	103.1 ± 14.6 (75-129)	p<0.001*

A higher percentage of VF defects were found in group A with superficial drusen (N=29, 90.6%) compared with group B with buried drusen (N=20, 71.4%), but without a statistically significant difference (p=0.113). The type of VF defect differed between groups (p=0.020). Regarding group A, VF constriction was the most frequent VF defect (N=15, 51.7%), followed by arcuate scotoma (N=9, 31%), blind spot enlargement (N=6, 20.7%), unspecific defects (N=2, 6.9%), and nasal step (N=1, 3.4%). Regarding group B, arcuate scotoma (N=7, 35%) and unspecific defects (N=6, 30%) were the most frequent types; other defects included VF constriction (N=4, 20%), blind spot enlargement (N=3, 15%), and nasal step (N=2, 10%).

## Discussion

ONHD is strongly related to VF defects, with most patients showing VF alterations [2,8,12,13]. In our study, VF defects were present in most eyes with ONHD (N=49, 81,7%). The types of defects identified included VF constriction, arcuate scotoma, blind spot enlargement, unspecific defects, and nasal step. These abnormalities have also been commonly described by other authors, in variable proportions, in patients with ONHD [2,5,8,12-14].

In the present study, the VF defect type significantly differed between eyes with superficial ONHD and eyes with buried ONHD. Buried ONHD showed a tendency for less frequent and more localized, unspecific VF defects. Similar studies have also found a higher frequency of VF defects in superficial ONHD [12,13]. Lee KM et al. studied the factors associated with different VF defects and found that a larger drusen height was a risk factor for an enlarged blind spot, and a thinner peripapillary RNFL thickness was associated with other VF defects [15].

In our study, eyes with superficial ONHD had a more pronounced functional loss (worse perimetric MD, VFI, and PSD indices) and more severe structural damage (worse peripapillary RNFL and GCC thinning) than eyes with buried ONHD. Other studies have also compared anatomical and functional alterations between superficial and buried ONHD with similar results. Gili P et al. found a significantly thinner peripapillary RNFL in superficial drusen compared with controls, in all quadrants except for the temporal quadrant [6]. There were no significant differences between buried drusen and controls. VF defects were more often observed in superficial ONHD patients (58%) compared with buried ONHD patients (52%). The quantitative analysis with perimetric MD and PSD indices also found worse functional impairment in superficial ONHD [2]. Malmqvist L et al. compared 109 eyes with superficial ONHD and 40 eyes with buried ONHD [11]. Regarding the presence of VF defects, there was a significant difference between superficial (88.3%) and buried (54.6%) ONHD. More prominent RNFL thinning and VF loss were noted in eyes with superficial ONHD. There was also a correlation between mean peripapillary RNFL thinning and the frequency and extent of VF defects, as measured by perimetric MD [11].

Additionally, we also observed that patients with superficial ONHD were significantly older than patients with buried ONHD. On one hand, the age-related decrease in RNFL thickness must be taken into consideration when studying the ONHD-related alterations, as worse structural parameters are partially explained by this expected age-related decline. On the other hand, it corroborates the presumed evolution of ONHD over time already described in the literature. It is known that buried ONHDs, which are more prevalent in younger patients, tend to progress to a superficial location and become calcified with age. The progressive drusen growth and increase in number, as well as the age-related thinning of the overlying RNFL, also contribute to ONHD becoming more visible with age [3]. VF defects have been shown to increase with age as the ONHD becomes more superficial [2,16]. The transition phase occurs mostly during adolescence, during which VF defects may rapidly progress [2,4]. It has been postulated that the gradual calcium accumulation produces compression of the optic disc close to the axons of the ganglion cells, leading to their death, and, consequently, to progressive VF loss [2]. This study corroborates that functional and structural impairment is more prominent in eyes with superficial ONHD, possibly because calcification leads to greater axonal damage [3,11].

The limitations of our study include its retrospective nature, which implies some missing data, and the small sample size. In this study, we included eyes with bilateral disease, which can influence the results. Patients were followed in a regular clinical setting without strict protocols for treatment and follow-up. However, this study tries to reflect our experience in everyday clinical practice.

## Conclusions

Most patients with ONHD present VF defects, although without significant impairment in visual acuity. The VF defect differs with the type of ONHD, with buried ONHD showing less frequent and more localized, unspecific VF defects. Buried ONHD is more prevalent in younger patients, progressing to a superficial location and becoming calcified with age. Functional and structural impairment is more evident in eyes with superficial ONHD, possibly because the presence of calcification leads to greater axonal damage.
